# Assessment of knowledge, attitude, and practices regarding the relationship of obesity with diabetes among the general community of Pakistan

**DOI:** 10.1016/j.heliyon.2024.e29081

**Published:** 2024-04-04

**Authors:** Arooj Kiran, Naseer Ali Shah, Shujaul Mulk Khan, Haroon Ahmed, Muhammad Kamran, Beenish Khan Yousafzai, Zeeshan Ahmad, Sunghoon Yoo, Heesup Han, Ibrahim Alasqah, António Raposo

**Affiliations:** a Department of Biosciences, COMSATS University Islamabad, Islamabad, Pakistan; b Department of Plant Sciences, Quaid-i-Azam University Islamabad, Pakistan; c Pakistan Academy of Sciences Islamabad, Pakistan; d International Society of Ethnobiology, Marrakech, Morocco; e Department of Obstetrics & Gynecology, MTI-Ayub Teaching Hospital, Abbottabad, Pakistan; f Hanmoo Convention (Oakwood Premier), 49, Teheran-ro 87-gil, Gangnam-gu, Seoul 06164, South Korea; g College of Hospitality and Tourism Management, Sejong University, 98 Gunja-Dong, Gwanjin-Gu, Seoul 143-747, South Korea; h Department of Public Health, College of Applied Medical Sciences, Qassim University, Buraydah, 51452, P.O. Box 6666, Saudi Arabia; i School of Health, University of New England, Armidale, NSW, 2351, Australia; j CBIOS (Research Center for Biosciences and Health Technologies), Universidade Lusófona de Humanidades e Tecnologias, Campo Grande 376, 1749-024, Lisboa, Portugal

**Keywords:** Attitude, Knowledge, Obesity, Pakistan, Practices, Diabetes Mellitus

## Abstract

This study sought to evaluate the influence of knowledge, attitude, and practices assessment on diabetes related to obesity in Pakistani society. Data was collected both through door to door and online survey approaches from 518 participants by using a pre-validated questionnaire. A total of 15.6% were underweight, 61.2% were normal weight, 17.2% were overweight and 5.8% were in obesity class I and 2.9% were classified as obesity class II. The co-occurrence of obesity and diabetes was 29% (n = 22) among diabetic individuals (n = 84). A majority of the residents (59.1%) were from cities. While 94% of the participants responded to what obesity was, 83.8% knew what diabetes was. Fast food, soft drinks, and mayonnaise were deemed to be healthy by 75.1% of the respondents. Obesity was viewed as a disease by 94.8%, a major health issue by 78.2%, and a weight-loss necessity by 44.6% of participants. Only 24.9% exercised every day, and 23.9% engaged in any physical activity daily. The majority of respondents (50.6%) never tried to lose weight and 23.2% ate junk food daily. The sociodemographic variables showed that the age ranges of 25–34 years (*P* < 0.001; OR 0.531), 45–54 years (*P* < 0.05; OR 0.527), and urban residency (*P* < 0.001; OR 0.128) had a significant association with knowledge. The factors of urban residency (*P* < 0.001; OR 3.996), being unmarried (*P* < 0.001; OR 1.95), and having an income of 51,000–70,000 (*P* < 0.001; OR 11.29) showed a very highly significant association with a good attitude regarding the relationship of obesity with diabetes (P < 0.05). Similarly, practices of the participants showed significant association with BMI range of 18.5–24.9 and 25–29.9 (*P* < 0.001)*.* Our study revealed significant knowledge and understanding of the relationship between obesity with diabetes. However, it was observed that majority of respondents exhibited fundamental knowledge regarding obesity and diabetes, there was a notable absence of understanding regarding crucial elements, such as the significance of maintaining a healthy body weight, participating in physical activity, and implementing appropriate dietary strategies for weight control. We recognize the necessity for education initiatives and strongly encourage them to assist individuals in managing diabetes resulting from obesity.

## 1 Introduction

Obesity is a rising epidemic in developing nations, represents a metabolic health concern of universal impact [[1], [2], [3]]. It is considered as one of the most crucial public health concerns of the 21st century [4]. One of the factors that can contribute to obesity is the consumption of excessive food combined with a sedentary lifestyle [5]. The prevalence of the obesity in Nepal has increased both in males and females over the past 30 years, rising from 7.9% to 18.2% and 6.9%–16.7%, respectively [6]. The percentage of men in Spain who were overweight was 44.31% compared to 30.04% of women in 2017 [5]. Diabetes is a chronic condition in which the body either does not make enough insulin or uses it insufficiently [7]. Uncontrolled diabetes-related hyperglycemia (high blood sugar) has been linked to the decline of numerous organs and bodily functions, including blood vessels and neurons [5]. Type 2, the most prevalent type of diabetes, accounts for 90% of all cases in Finland [8]. Due to shifts in lifestyle habits and prolonged lifespans, the incidence rate is rapidly increasing [9]. The World Health Organization (WHO) now considers both obesity and diabetes mellitus (DM) to be an epidemic due to the increasing incidence and prevalence [10]. Diabetes was identified as the cause of 1.6 million fatalities in adults in 2014, accounting for 8.5% of all diagnoses. Its prevalence has sharply increased in low- and middle-income countries [11]. According to the projections by the International Diabetes Federation, the number of people worldwide with type 2 diabetes (DM2) is expected to reach 642 million by the year 2040. This significant increase is anticipated from an estimated 415 million people with DM2 in 2018.

Individuals with obesity, are at a higher risk of developing various illnesses and health conditions, including type 2 diabetes (T2D), heart disease, high blood cholesterol, stroke, osteoarthritis, and respiratory problems. These health risks are associated with excess body weight and adipose tissue, which can lead to metabolic disturbances and increased strain on cardiovascular and musculoskeletal systems. Maintaining a healthy weight through balanced nutrition and regular physical activity is essential to reduce the likelihood of these ailments and improve overall well-being [[12], [13], [14]]. The existing epidemic of type 2 diabetes is widely regarded to be primarily driven by obesity and being overweight [15]. Recognizing the close relationship between these two conditions, “diabesity” has become a catch-all term for both conditions [16]. Similar to adults, children with high BMIs are at higher risk of developing insulin resistance [[17], [18], [19]]. However, studies have shown that overweight children have a higher likelihood of becoming overweight adults as they gets older [20]. The possibility that these kids will continue to be overweight as adults is higher for those with high BMIs between the ages of two and five. Consequently, the obesity epidemic seriously threatens public health [21]. In 2016, there were around 1.9 billion adults with obesity globally [22,23]. In 2016, 13% of overweight and 39% of adults with obesity were over 18. The World Health Organization predicts that the number of overweight children under the age of five is expected to reach 40 million [24]. This concerning statistic highlights the global issue of childhood obesity, which can have significant implications for the children's health and well-being in the long term. Early intervention and adopting healthy lifestyle practices are essential to address this growing public health concern and promote better health outcomes for children [25]. Malnutrition is thought to have been pervasive among Pakistan's population for a long time. However, regarding population size and obesity rates, Pakistan ranked ninth [26].

As per the International Diabetes Federation (IDF), 33 million people are affected from diabetes [27]. Prevalence of type 2 diabetes in Pakistan is gradually increasing and is currently at an alarming level of 13.7% with 13.8 millions of adults [28,29]. Moreover, Pakistan is ranked forth globally in prevalence of diabetes [30]. It is estimated that prevalence of T2D in pakistan will rise to 15% in 2040 [31]. Pakistan has a concerningly high obesity rate, with estimates indicating a prevalence of 27.85% among the population [32]. When compare with statistics of other countries, Pakistan is facing a higher prevalence of T2D. Brazillian and Mexico population has prevalence of 8.8% [33], much lower than Pakistan [28,31]. Type 2 diabetes and obesity have a significant relationship [34,35] as body mass index (BMI) and type 2 diabetes risk and severity correlate positively [36,37]. The astonishing seven-fold increased risk encountered by the obesity is shocking compared to the overweight, who have a three-fold greater risk of developing diabetes [38]. Because of the metabolic changes brought on by obesity, adipose tissue might contribute to the emergence of insulin resistance by secreting chemicals such as non-esterified fatty acids, glycerol, hormones, and pro-inflammatory cytokines [[39], [40], [41]]. Blood sugar levels spiral out of control when insulin resistance and pancreatic islet cell loss occur simultaneously. Predicting the likelihood of developing type 2 diabetes and tracking its progression requires monitoring for shifts in cell function [21,42]. Addressing health concerns associated with obesity presents a substantial obstacle for developing countries such as Pakistan. Several studies have revealed a correlation between obesity and diminished job performance, as individuals with a high body mass index experience limitations in mobility and an increased vulnerability to conditions such as type 2 diabetes. This study aims to assess the knowledge, attitudes, and practices (KAP) regarding the association between obesity and diabetes among residents in Pakistan. The main aim of this study is to acquire a more comprehensive understanding of the level of awareness, comprehension, and behavioral tendencies related to this significant health issue.

## 2 Materials and methods

### 2.1 Study site

**Fig. 1:**
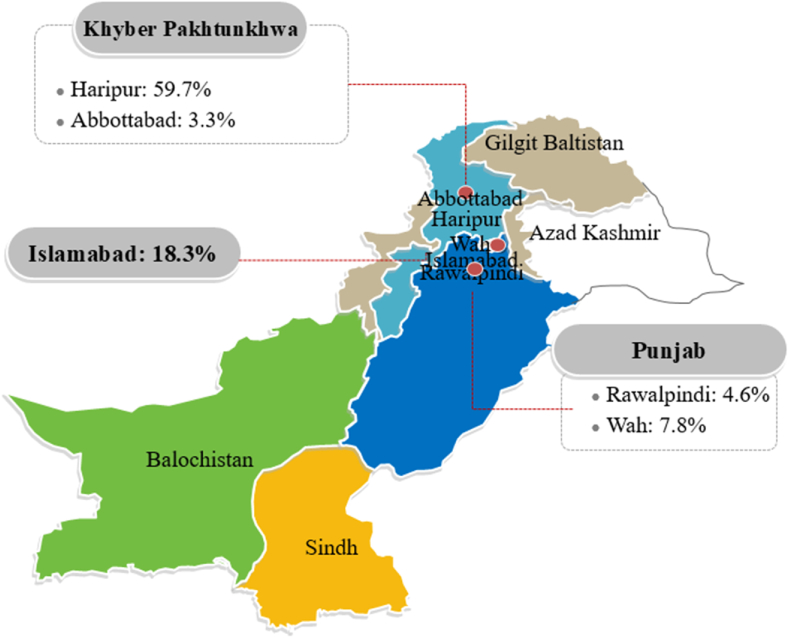
Map of Pakistan the study area where the knowledge, attitude, and practices (KAP) survey was conducted. The study area is shown in red color along with the collected response percentage. (For interpretation of the references to color in this figure legend, the reader is referred to the Web version of this article.)

The current study was carried out in Federal capital Islamabad and Haripur District of Khyber Pakhtunkhwa (KP). Islamabad is situated on the northern edge of the Potohar plateau, at an elevation of 540 m, and has a population of 2.015 million people [43]. Haripur is a district in Pakistan's Khyber Pakhtunkhwa province. It is located in the Hazara region and is bordered to the north by Abbottabad district, by Islamabad Capital Territory to the east, by Attock district of Punjab to the southeast, and by Swabi district of Khyber Pakhtunkhwa to the west (Fig. 1).

### 2.2 Study design

This cross-sectional study was carried out to assess the knowledge, attitude, and practices (KAP) regarding the relationship of obesity with diabetes among the general community of Pakistan. A standard pre-validated questionnaire (Supplementary Table 1) was designed from a literature review in English and translated into the national language, Urdu. Both qualitative and quantitative data were collected to assess the relationship. The responses were collected both through an online survey and door to door interviews.

### 2.3 Sample size calculator

The sample size was calculated using the “Rao soft” online sample size calculator under the assumptions of a 95% confidence interval, a 5% margin of error, Z of 1.96, and at response distribution of 50% [44]. The required sample size was 385, and we collected data from 518 participants during the current study.

### 2.4 Study questionnaire

As a survey tool, we used a pre-validated Knowledge, Attitude and Practices (KAP) online questionnaire using Google Forms employing the body of existing research, the preexisting data, and our prior experience and understanding in the field [1,12,26,45,46]. A four-part questionnaire was designed with both positive and negative sentences. The questionnaire had questions about sociodemographic characteristics (n = 12), 19 knowledge (n = 19), attitude (n = 15), and practices (n = 20). Likert scale was used to assess the Knowledge, Attitude, and Practices of the participants. The sum of the responses was calculated. Score of 1–2 was considered as poor, while 3–4 was considered as good (Saleh et al., 2012). Specific parameters were used to measure the BMI of the participants.

### 2.5 Data collection and quality control

The pre-validated questionnaire was circulated both in English and the national language Urdu. We collected both qualitative and quantitative data through a door-*to*-door survey and by disseminating the online questionnaire to the target population. Training was arranged for the data collector and field supervisor. They were briefed about inclusion and exclusion criteria, ethical issues, data privacy, the art of data collection, interviewing, and about getting permission before the collection of data. Post graduate students (n = 4) and a field supervisor (n = 1) received this training. A pilot study was conducted by recruiting 50 participants. To ensure the data collection procedure, review was conducted twice a week by the supervisor and a principal investigator. Only completely filled forms were included in the current study. Interviews were conducted to collect the data from illiterate individuals. An informed consent was taken from the participants before collection of data and their identities were kept confidential.

### 2.6 Dependent & independent variables

Knowledge, attitude, and practices were taken as dependent variables. Sociodemographic chracteristics (gender, age, residency, income status etc), BMI, and status of diabetes were taken as independent variables.

### 2.7 Data analysis

For storage and sorting purposes, aft Excel file was used, and SPSS 24.0 was used for statistical analysis. The frequency and percentages of correct responses were calculated using descriptive statistics. To investigate the relationship between two categorical variables, a Chi-square test was applied. Binomial logistic regression analysis was used as dichotomous outcome variables. Dependent variables, like knowledge, attitudes, and practices of participants, were selected on basis of desired outcome. Independent variables included sociodemographic characteristics, age and marital status etc. To evaluate the correlation between independent predictors and outcome variables, odds ratios with 95% confidence intervals were calculated. The significance level was set at 0.05.

## 3 Results

The sociodemographic factors indicates gender, age, education, BMI, residency, marital status, and income. 59.1% of the participants were from urban region compared to rural areas (40.9%). Most respondents were males (53.1%) compared to females (46.9%). Of the 518 respondents, 15.6% were underweight (BMI <18.5), 61.2% had normal-weight, and 5.8% were in obesity class I and 2.9% were classified as obesity class II. Many respondents had a monthly income of 31,000–51,000 PKR (Pakistani rupees), while 10% were illiterate (Supplementary Table 2).

**Fig. 2:**
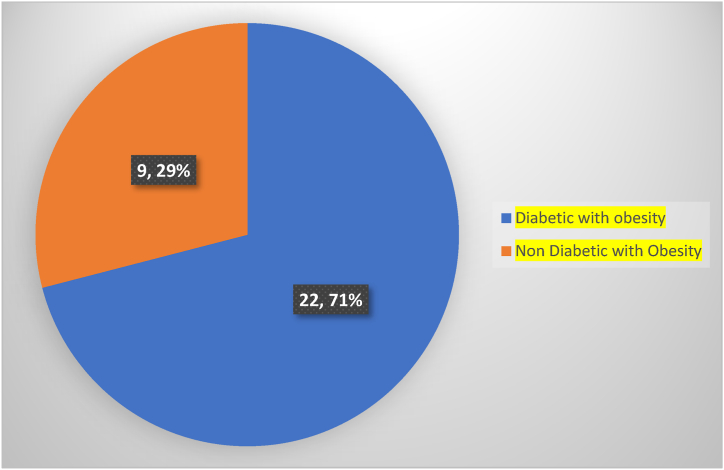
Co-ocurrence of obesity & Diabetes.

The majority of the respondents (94%) were aware of obesity, 83.8% reported that they knew about diabetes, and more than half (56.4%) responded that they knew of the relation of obesity to diabetes. This study found that 50.8% of respondents knew that patients with obesity usually have diabetes. About 69.7% responded that they knew their ideal body weight, and 33.6% knew about normal BMI. When asked about the BMI of overweight and obesity, only 30.5% of the respondents indicated that a person with a BMI of 25–29.9 is considered overweight and a person with a BMI of 30.0 or higher is deemed with obesity. According to this study, 79.9% of participants were aware of what causes obesity. When asked about energy requirements, more than half (56.9%) of the respondents knew about energy requirements. Contrary to the majority's (75.1%) claim that fast food, soda, and mayonnaise are good for the body. The majority of participants (74.9%) were aware that obesity and family history are the main contributors to diabetes. However, more than half of respondents (51.8%) did not know that the diabetes risk is highest in people with obesity, and only 27.8% knew that those with a BMI of 30 kg/m^2^ had the highest risk of developing diabetes. Only 39.2% knew obesity-related metabolic changes that are the causes of developing diabetes (Table 1). We assessed the co-occurrence of obesity and diabetes. Among 84 diabetic individuals, 71% (n = 62) of the participants were diabetic without obesity, while, 29% (n = 22) were both with obesity and diabetes (Fig. 2), and 9 participants were with obesity without diabetes.

**Table 1:** Responses regarding knowledge of obesity among the participants.

Variables	Scale	No. (N)	Frequency (%)
Do you know the term “obesity”?	Yes	487	94.0
	No	31	6.0
Do you feel the need to lose weight?	Yes	231	44.6
	No	287	55.4
Do you know what the term “diabetes” means?	Yes	482	83.8
	No	36	16.2
Are you a diabetic patient?	Yes	84	16.2
	No	434	83.8
Do you think those who are with obesity also have diabetes?	Usually	263	50.8
	Maybe	235	45.4
	No	20	3.9
Did you know that obesity is associated with diabetes?	Yes	292	56.4
	Maybe	158	30.5
	No	50	9.7
	Don't know	18	3.5
Did you know that obesity is also associated with hypertension, stroke, cardiovascular diseases, and some cancers?	Agree	285	55.0
	Maybe	99	19.1
	Don't know	121	23.4
	Disagree	13	2.5
Have you heard the methods to determine obesity such as BMI, waist circumference, and waist–hip ratio (WHR) etc.?	Yes	260	50.2
	No	258	49.8
Do you know what your ideal body weight is?	Yes	361	69.7
	No	157	30.3
Did you know that a person with a BMI of 18.5–24.9 is considered healthy?	Yes	174	33.6
	Maybe	101	19.5
	Don't know	193	37.3
	No	50	9.7
Is a person with a BMI of 25–29.9 considered overweight, and a person with a BMI of 30.0+ considered obese?	Yes	158	30.5
	Maybe	159	30.7
	Don't know	158	30.5
	No	43	8.3
Did you know that physical inactivity, eating too much fat, sleep routine, hormone disorders, stress, and anxiety causes of obesity?	Yes	414	79.9
	Maybe	79	15.3
	No	25	4.8
Do you know how much energy is required for the ideal body weight?	Yes	223	56.9
	No	295	43.1
Are fast food, soft drinks, mayonnaise healthy food for the body?	Yes	389	75.1
	No	129	24.9
Did you know having a blood glucose level less than 140 mg/dL (7.8 mmol/L) is normal?	Yes	255	49.2
	Maybe	117	22.6
	Don't know	100	19.3
	No	46	8.9
Did you know diabetes risk is highest in obese people with a BMI of 30 kg/m^2^?	Yes	144	27.8
	Maybe	106	20.5
	Don't know	219	42.3
	No	49	9.5
Did you know that family history and obesity are the major causes of diabetes?	Agree	388	74.9
	Maybe	71	13.7
	Don't know	47	9.1
	Disagree	12	2.3
Are you a smoker?	Yes	122	23.6
	No	396	76.4
Do you agree that the obesity is also thought to trigger changes to the body's metabolism, leading to reduced insulin sensitivity and, alternatively, developing into diabetes?	Agree	203	39.2
	Maybe	57	11,0
	Don't know	245	47.3
	Disagree	13	2.5

The majority of the respondents (94.8%) reported that obesity is a disease, 86.9% responded that average weight is necessary for good health, 78.2% reported that obesity is a severe health problem, and 45.6% responded that even a small amount of weight loss could have important health advantages, such lowering the risk of acquiring diabetes. The majority of respondents (86.9%) reported that people with obesity must try to lose weight, and 79.5% reported that regular exercise helps in losing weight. The majority of respondents (71%) said that being overweight increases a person's risk of developing diabetes, 60.5% said that relatives of diabetic patients should be aware of obesity, and 83.6% said that they support taking preventative measures to avoid obesity and diabetes. We discovered that 57.9% of the respondents said their body weight is under control, and 50.8% said regular exercise helps to control diabetes and lower blood sugar levels. 47.9% said that medication is not more important than food and exercise to control diabetes and obesity, while approximately 59.7% noted that non-drug treatments are available for diabetes (Table 2).

**Table 2:** Frequency of attitude regarding obesity among the participants.

Variables	Scale	No. (N)	Frequency (%)
Do you think obesity is a disease?	Yes	491	94.8
	No	27	5.2
Do you think normal body weight is necessary for good health?	Yes	450	86.9
	Maybe	61	11.8
	No	7	1.4
Do you consider obesity a serious health problem?	Yes	405	78.2
	Maybe	72	13.9
	Don't know	34	6.6
	Disagree	7	1.4
Do you think minor weight loss can produce important health benefits, such as lower the risk of developing diabetes, heart disease, and types of cancer?	Yes	236	45.6
	Maybe	223	43.1
	Don't know	35	6.8
	No	24	4.6
Do you think obese people must try to lose weight?	Yes	450	86.9
	Maybe	60	11.6
	No	8	1.5
Do you think regular exercise helps you in losing weight?	Yes	412	79.5
	Maybe	98	18.9
	No	8	1.5
Have you ever tried to lose weight?	Yes	245	47.3
	No	273	52.7
Do you exercise daily?	Yes	129	24.9
	Occasionally	201	38.8
	No	188	36.3
Do you think being overweight increases a person's risk of developing diabetes?	Yes	365	70.5
	Maybe	129	24.9
	Don't know	7	1.4
	No	17	3.3
Are you following a planned and healthy diet?	Yes	433	83.6
	No	85	16.4
Do you care about your body shape?	Yes, very much	299	67.7
	Somewhat	138	26.6
	Not at all	81	15.6
Do you think your body weight is under control?	Yes	300	57.9
	Maybe	112	21.6
	No	106	20.5
In your opinion, a family member of a diabetic patient should be aware of the facts regarding obesity?	Yes	314	60.6
	Maybe	183	35.3
	No	21	4.1
Do you believe in precautionary measures to prevent the obesity and diabetes?	Strongly agree	232	44.8
	Agree	253	38.8
	Don't know	17	3.3
	Disagree	16	3.1
Do you believe diabetes is caused by obesity?	Yes	305	58.9
	Maybe	194	37.5
	No	19	3.7
Do you think smoking is associated with a higher risk of abdominal obesity or belly fat, which is one of the risk factors for diabetes?	Agree	152	29.3
	Maybe	44	8.5
	Don't know	301	58.1
	Disagree	21	4.1
Do you believe in non-drug treatments of diabetes?	Yes	309	59.7
	Somewhat	144	27.8
	No	65	12.5
Do you think that regular exercise lowers blood glucose levels and cures diabetes?	Yes	263	50.8
	Maybe	114	22.0
	Don't know	131	25.3
	No	10	1.9
Do you think medication is more important than diet and exercise to control diabetes and obesity?	Agree	149	28.8
	Neutral	58	11.2
	Don't know	63	12.2
	Disagree	248	47.9
Did you know that diabetic patients should take special care when cutting the toenails?	Yes	495	95.6
	No	23	4.4

Only 23.9% of the respondents reported that they daily participated in any physical activity, while 36.7% responded that they do not participate in any physical activity. Among all the respondents, more than half (50.6%) never tried to lose weight, 22% had tried to lose weight by more than two times, and 16.4% had only once tried to lose weight. 85.9% never visited a weight counseling seminar or workshop. About 48.3% had their blood sugar tested. Lastly, we found that 35.7% of reported that treating obesity-related diabetes is very costly (Table 3).

**Table 3:** Practices regarding obesity among the participants.

Variables	Scale	No. (N)	Frequency (%)
How frequently do you check your body weight?	Every 1–3 months	195	37.6
	Every 4–6 months	138	26.6
	Every 7–9 months	68	13.1
	Every 10–12 months	100	19.3
	After 1 year	7	1.4
	Never	10	1.9
How many times have you tried to lose weight?	More than twice	114	22.0
	Twice	57	11.0
	Once	85	16.4
	Never	262	50.6
How much exercise do you do to lose weight?	200 min a week	40	7.7
	150 min a week	36	6.9
	100 min a week	63	12.2
	50 min a week	95	18.3
	30 min a week	156	30.1
	None	128	24.7
Have you visited a weight counseling seminar or workshop?	Yes	73	14.1
	No	445	85.9
Do you participate in any physical activity?	Yes	124	23.9
	Sometimes	204	39.4
	No	190	36.7
What methods do you think are effective for your long-term weight loss?	Exercise	38	7.3
	Healthy diet	41	7.9
	Physical activity	135	26.1
	Healthy diet, exercise	29	5.6
	Healthy diet, physical activity	92	17.8
	Exercise, physical activity, healthy diet	72	13.9
	Physical activity, exercise	106	20.5
	No one	5	1.0
How frequently do you eat junk food?	Never	88	17
	Occasionally	151	29.2
	Once a month	6	1.2
	Once a week	10	1.9
	More than twice a day	65	12.5
	Twice a day	78	15.1
	Once a day	120	23.2
Do you eat sweets after a meal?	No	72	13.9
	Rarely	156	30.1
	Sometime	192	37.1
	Daily	98	18.9
Do you eat more fruits and vegetables?	Yes	471	90.9
	No	47	9.1
Have you had your blood sugar tested ?	Yes	250	48.3
	No	268	51.7
What method do you mainly use for testing your blood sugar levels?	Blood or urine test	269	51.9
	None	119	23.0
	Not available	130	25.1
Have you experienced any side effects of medication that you know of?	No	247	47.7
	Yes	121	23.4
	Not available	150	29.0
Do you take any additional nutritional supplements?	Herbal supplements or Vitamins	91	17.6
	No	427	82.4
If you are diabetic, how often do you visit a doctor for your diabetes?	Monthly	1	0.2
	5 or more times a year	18	3.5
	2-3 times a year	33	6.4
	Once a year	129	24.9
	Never	150	29.0
	Not available	187	36,1
Do you think treating obesity-related diabetes is very costly?	Yes	185	35.7
	Maybe	241	46.5
	Don't know	26	5.0
	No	66	12.7

By using binomial logistic regression analysis, we determined the association of knowledge, attitude, and practices with sociodemographic characteristics link to this disease. Each collection of independent variables is used as a reference category from sociodemographic components. The sociodemographic factors determined that age groups from 25 to 34 years (*P* < 0.001; OR 0.531), 45–54 years (*P* < 0.05; OR 0.527), and urban residency (*P* < 0.001; OR 0.132) had a highly significant association with knowledge. Factors of qualification, including matric-level (*P* < 0.001; OR 0.0147), intermediate-level (*P* < 0.001; OR 0.0078), graduate-level (*P* < 0.001; OR 0.00831), postgraduate-level (*P* < 0.001; OR 0.00392), factors of marital-status including unmarried (*P* < 0.001; OR 0.338), and factors of income those having 51,000–70,000 (*P* < 0.01; OR 0.163), indicated a very strong correlation with the understanding of the connection between diabetes and obesity. With the dependent variable attitude, we obtained p-values of factor age from groups 45–54 years (*P* < 0.01; OR 2.58); 65–74 years (*P* < 0.05; OR 3.58), factors of qualification illiterate-level (*P* < 0.001; OR 0.014); primary-level (*P* < 0.01; OR 9.4561); middle-level (*P* < 0.001; OR 23.5926); matric-level (*P* < 0.001; OR 28.4058); intermediate-level (*P* < 0.001; OR 39.3922), graduate-level (*P* < 0.001; OR 69.5214), -post-graduate-level (*P* < 0.001; OR 106.1667) indicated a positive attitude and had a significantly significant correlation with attitude. Urban residency factor (*P* < 0.001; OR 3.996), unmarried (*P* < 0.001; OR 1.95), and factors of income those having 51,000–70,000 (*P* < 0.001; OR 11.29) showed very highly significant association (P < 0.05) with the good attitude regarding the relationship of obesity with diabetes.

Practices were determined from several factors, including males (*P* < 0.001; OR 2.36), age groups from 35 to 44 years (*P* < 0.001; OR 0.097); 45–54 years (*P* < 0.001; OR 0.097); 55–64 years (*P* < 0.001; OR 0.173); 65–74 years (*P* < 0.01; OR 0.196), and factors of BMI ranges from 18.5 to 24.9 (*P* < 0.001; OR 0.173), and from 25 to 29.9 (*P* < 0.001) was significantly associated with practices. Factors of qualification, including graduate-level (*P* < 0.001; OR 0.306); postgraduate-level (*P* < 0.01; OR 0.246), urban residency (*P* < 0.05; OR 0.65), and unmarried (*P* < 0.001; OR 0.35), showed significant association (P < 0.05) with practices status regarding the relationship of obesity with diabetes. All other sociodemographic factors showed a non-significant association with knowledge, attitude and practices regarding the relationship between obesity with diabetes (Table 4).

**Table 4:** Association of knowledge, attitude, and practices with socio-demographics variables regarding obesity among studied participants.

Variables	Category	Good	Poor	OR (95% CI)	P-value	Good	Poor	OR (95% CI)	P-value	Good	Poor	OR (95% CI)	P-value
		Knowledge				Attitude				Practices			
Gender	Male	167	108	0.708 (0.499–1)	0.053	184	91	1.11 (0.770–1.59)	0.582	184	91	2.365 (1.657–3.38)	<0.001
	Female (Base)	127	116	–	–	157	86	–	–	112	131	–	–
Age	18-24 (Base)	94	97	–	–	117	74	–	–	140	51	–	–
	25–34	93	51	0.531 (0.341–0.828)	0.005	89	55	1.02 (0.656–1.60)	0.919	94	50	0.6849 (0.4282–1.095)	0.114
	35–44	37	24	0.629 (0.350–1.130)	0.121	41	20	1.30 (0.705–2.38)	0.403	30	31	0.0976 (0.0507–0.188)	<0.001
	45–54	46	25	0.527 (0.30–0.925)	0.026	57	14	2.58 (1.340–4.95)	0.005	15	56	0.0976 (0.0507–0.188)	<0.001
	55–64	12	19	1.534 (0.706–3.335)	0.280	20	11	1.15 (0.521–2.54)	0.729	10	21	0.1735 (0.0765–0.393)	<0.001
	65–74	12	8	0.646 (0.253–1.651)	0.362	17	3	3.58 (1.015–12.65)	0.047	7	13	0.1962 (0.0741–0.519)	0.001
BMI	<18.5 (Base)	44	37	–	–	53	28	–	–	69	12	–	–
	18.5–24.9	193	124	0.764 (0.467–1.25)	0.284	209	108	1.02 (0.612–1.71)	0.933	158	159	0.173 (0.09–0.33)	<0.001
	25–29.9	42	47	1.331 (0.728–2.43)	0.353	62	27	1.21 (0.638–2.31)	0.556	39	50	0.136 (0.064–0.285)	<0.001
	30–39.9	15	16	1.235 (0.36–3.2)	0.596	17	14	0.64 (0.331–1.67)	0.351	30	1	5.621 (0.492–38.462)	0.19
Education	Illiterate (base)	1	51	120.393 (16.270–890.885)	–	3	49	0.0144 (0.0042–0.0486)	–	38	14	3.269 (1.670–6.40)	–
	Primary	3	27	21.246 (6.211–72.673)	0.141	11	19	0.1360 (0.0598–0.3089)	–	18	12	1.806 (0.828–3.94)	0.223
	Middle	2	20	23.607 (5.352–104.125)	0.193	13	9	0.3394 (0.13540.8504)	0.001	11	11	1.204 (0.500–2.90)	0.059
	Matric	36	27	1.770 (0.989–3.168)	<0.001	40	23	0.4086 (0.2197–0.7597)	<0.001	52	11	5.693 (2.810–11.54)	0.224
	Intermediate	83	33	0.939 (0.568–1.551)	<0.001	82	34	0.5666 (0.3333–0.9632)	<0.001	72	44	1.971 (1.238–3.14)	0.167
	Graduate	144	61	–	<0.001	166	39	–	<0.001	93	112	–	<0.001
	Post-graduate	25	5	0.472 (0.173–1.291)	<0.001	26	4	1.5271 (0.5038–4.6289)	<0.001	12	18	0.803 (0.368–1.75)	0.004
Residency	Rural (Base)	62	150	–	–	101	111	–	<0.001	134	78	–	–
	Urban	232	74	0.132 (0.0888–0.196)	<0.001	240	66	3.996 (2.724–5.86)	–	162	144	0.655 (0.458–0.937)	0.021
Marital status	Married (Base)	114	146	–	–	152	108		<0.001	181	79	–	–
	Unmarried	180	78	0.338 (0.236–0.486)	<0.001	189	69	1.95 (1.34–2.82)	–	115	143	0.351 (0.245–0.503)	<0.001
Income/month	<10,000 (Base)	5	7	–	–	6	6	–	<0.001	8	4	–	–
	<30,000	59	95	1.150 (0.348–3.791)	0.818	79	75	1.05 (0.325–3.41)	–	97	57	0.851 (0.245–2.95)	0.799
	31,000–51,000	115	83	0.516 (0.1581–1.681)	0.272	129	69	1.87 (0.581–6.02)	0.931	124	74	0.838 (0.244–2.88)	0.779
	51,000–71,000	70	16	0.163 (0.0459–0.581)	0.005	79	7	11.29 (2.867–44.43)	0.294	36	50	0.360 (0.101–1.29)	0.116
	>71,000	45	23	0.365 (0.1043–1.278)	0.115	48	20	2.40 (0.690–8.34)	<0.001	31	37	0.419 (0.115–1.52)	0.187

Parameter significant at P < 0.05.

## 4 Discussion

The prevalence of obesity among adults has become a significant concern within the realm of public health in recent decades. The prevalence of overweight and obesity, particularly those residing in urban areas, has exhibited a notable upward trend. Females within this demographic group tend to occupy a greater proportion of the overweight and obesity compared to their male counterparts [47,48]. According to the World Health Organization, over 1.9 billion people worldwide were overweight in 2016, with 650 million of those with obesity. The prevalence of obesity increased globally between 1975 and 2016 [49]. According to the World Obesity Federation, 21.8% of women in Pakistan are suffering from obesity, 30.4% are overweight, 12.1% of men are with obesity, and 25.1% are overweight. The rate of obesity was 4.3% among male and 5.1% in females, while the rate of being overweight was 3.3% in males and 4.0% in females [50].

While Pakistan is now ranked 3rd in terms of diabetes prevalence, behind China and India, the country has approximately 33 million individuals with the disease [51]. According to figures in the IDF Diabetes Atlas 10th edition, 537 million individuals (20–79 years) or 1 in 10, have diabetes, and this figure is expected to increase to 643 million by 2030 and 783 million by 2045 [52].

According to a study, type 2 diabetes mellitus affects 11.77% of the population in Pakistan. Males account for 11.20%, while females account for 9.19% [53]. In Sindh province, men have a higher prevalence (16.2 %), while females account for 11.70 %. In Punjab, males have a prevalence of 12.14 % and females have a prevalence of 9.8 %. Males comprise 13.3% of Baluchistan's population, while females comprise 8.9%. Males make up 9.2% of the population of Khyber Pakhtunkhwa (KPK), while females make up 11.60%. Type 2 diabetes mellitus is prevalent in Pakistan at a rate of 14.81% in urban areas and 10.34% in rural areas [54].

The widespread prevalence of obesity poses a significant hindrance to the fight against diabetes and its prevention. Enabling patients to assume the role of primary decision-makers necessitates furnishing them with comprehensive guidance to facilitate well-informed choices pertaining to both preventive measures and healthcare interventions. In order to enhance its effectiveness, patient education should be carefully tailored to align with the distinct characteristics of each individual, including their background, level of understanding, and proficiency. The theoretical framework known as the knowledge, attitude, and practices (KAP) model proposes that increasing knowledge about a particular health-related topic leads to an initial shift in perspective, which in turn facilitates changes in behavior. However, there is a significant lack of research in examining the knowledge, attitudes, and practices (KAP) paradigm in relation to diabetes resulting from obesity. A study conducted in Japan included 2158 respondents, 1031 males and 1127 females aged 18–50 years (97.2% 30 years). 28.1% of participants were overweight or with obesity, 12.7% were underweight, and 59.2% had a normal BMI. The males had a greater average BMI than the females [55,56]. In Asian countries, a majority of patients develop diabetes with lower BMI than Western countries [57]. A study found that in the Asian population, the reason for lower BMI and risk of diabetes is due to Asian specific fat depots [58]. According to one study, being overweight (BMI ≥ 30 kg/m2) is becoming more common in Western countries and is often linked to other health problems like type 2 diabetes mellitus [59]. South Asians are at higher risk of developing diabetes with lower avaergae BMI than many other racial & ethnic groups [60].

The findings of the study revealed a moderate level of participant knowledge regarding the interconnectedness of obesity and diabetes. A noteworthy portion of the respondents demonstrated awareness of obesity, with a majority also displaying familiarity with diabetes. Moreover, over half of the participants acknowledged an understanding of the correlation between obesity and diabetes. Additionally, a considerable number of respondents reported awareness of other health conditions linked to obesity. The study also observed that a significant proportion of participants, more than half, possessed knowledge about the association between obesity and diabetes in terms of its causal relationship. Furthermore, the survey results highlighted a prevalent understanding among the participants that genetics and excess fat are primary contributors to the onset of diabetes, aligning with widely recognized knowledge. A similar pattern of findings was noted in South Jordan, where approximately 37% of the 2158 participants disclosed a family history of diabetes, with a nearly equivalent percentage reporting their involvement in the care of a family member affected by the disease [55].

Our findings indicate that a significant proportion of the participants lacked awareness regarding the association between individuals with a body mass index (BMI) of 30 kg/m^2^ and the heightened susceptibility to diabetes. Furthermore, it was observed that over fifty percent of the respondents were unaware of the elevated risk of diabetes among individuals with obesity. A mere 39.2% of the participants demonstrated awareness of the obesity-induced metabolic alterations that serve as precursors for the onset of diabetes. A comparable cross-sectional study by Ref. [1] was conducted on a sample of 160 individuals diagnosed with type 2 diabetes at a tertiary healthcare facility located in Bangladesh. The researchers discovered that a majority of individuals lacked knowledge regarding optimal body weight, energy requirements, and methods of weight measurement. Furthermore, when queried about the nutritional value of various commodities, a significant proportion of respondents inaccurately categorized fast food, soft drinks, and mayonnaise as being among the most nutritious options. The development and progression of obesity are significantly influenced by the suboptimal dietary habits of patients. Existing evidence indicates that restricting the consumption of fat and sugar can effectively aid in the management of body weight and prevent the onset of these conditions. Another similar study, conducted among medical students in Faisalabad, Pakistan shows that among the 208 participants, 97% were familiar with the word obesity. The most commonly known method to determine obesity was the measurement of BMI. Majority of the paticipants (98%) knew about cardiovascular diseases and diabetes as potential ill effects of obesity, but social and psychological problems were lesser known. In terms of factors leading to obesity, eating too much fat, insufficient physical activity, genetic factors, and hormonal disorders were more commonly identified than stress, anxiety and depression, and high socioeconomic status. Two-thirds of the participants were able to identify the correct normal BMI range [46,61].

Most respondents agreed that obesity is a medical condition and that maintaining a healthy body weight is important in controlling and prevention of the disease. Most respondents agreed that those who are overweight should make an effort to reduce their weight, that exercising regularly is beneficial in this regard, and that the risk of developing diabetes increases with excess body fat. A prevailing consensus emerged from the data, with the majority of respondents indicating that individuals who are with obesity should actively pursue weight loss. Additionally, there was a widespread acknowledgment of the positive impact of regular exercise in facilitating weight reduction. Furthermore, a prominent perception among respondents was that the risk of developing diabetes escalates with an increase in body weight. This study discovered that more than half of respondents said their body weight was under control and that regular exercise helped them reverse their diabetes. However, some argued that even a small amount of weight loss could positively impact one's health, lowering their risk of acquiring diabetes. According to a study conducted in Pakistan among university faculty members about the benefits of physical activity in combating obesity, nearly 58.70% agreed or strongly agreed that obesity is a very serious condition. Over 55% of university staff members displayed unfavorable attitudes toward cutting back on their eating and exercising [45]. Another similar study conducted among medical students in Faisalabad, Pakistan shows that among the 208 participants, the overall response in the attitude section was satisfactory. Almost all students (98%) were positive about being role models and maintaining a normal-weight. However, they noted that 20% of the population still does not recognize obesity as a disease and holds the widespread myth that it is genetic and therefore incurable [46]. Less than half of those surveyed said that to control diabetes and obesity, medicine is not more crucial than diet and exercise. The same study, which included 160 type 2 diabetic patients, was conducted in a Bangladesh tertiary care hospital. They concluded that the majority of respondents had a good opinion toward maintaining an ideal body weight and preventing or controlling obesity through diet and activity management. However, it was established that this attitude does not translate into actual practice. Only half of the respondents maintained a healthy diet and exercise regimen, and overall exercise was insufficient [1]. A similar study conducted in Bangladesh revealed notable disparities, with individuals working in the service sector having a significantly higher mean practice score. This disparity could be attributed to such issues as limited access to information or a lack of knowledge dissemination. As a result, this specific demographic necessitates targeted intervention efforts to address these potential gaps. Surprisingly, the practice score revealed a significant difference between the normal-weight and obesity groups. Notably, healthy behaviors were more prevalent among those who were normal-weight. Notably, the study discovered a gradual decrease in exercise duration as individuals' BMIs increased. This decrease in physical activity has been strongly linked to a pivotal role in the development of obesity. Improving patients' understanding of the underlying dynamics can help them develop a more positive outlook. This increased understanding can eventually lead to the adoption of healthier lifestyle choices, such as a more balanced diet and increased physical activity. Those with diabetes can potentially avoid the onset of additional complications by embracing these constructive practices, thereby fostering a healthier trajectory [1]. Another similar study conducted among medical students in Faisalabad, Pakistan shows that among the 208 participants, half the students had a habit of snacking after meals, and 20% did not participate in sports at all. Only about one-third of the population regularly engages in the activity of walking for pleasure [46]. Researchers in Pakistan found that only 15.22% of university workers regularly engage in physical activity (PA), despite previous research showing that it can significantly combat obesity. Only 2.2% of the 15.2% of the population who claimed to be engaged in PA did so four or more times per week, compared to 4.7% who did so weekly. In addition, 35.5% of men and 43% of women said that their work-related exhaustion and workload were the primary reasons why they did not engage in physical activity. One of the primary reasons people did not engage in physical activity was laziness (males: 41.3%; females: 36.6%). More than 64% of those engaged in diets designed to reduce obesity, including 68.6% of women and 61.9 of men, consumed fast, junk, sugary, fatty, or alcoholic beverages daily or twice weekly [21].

## 5 Conclusion

This study underscores a significant level of awareness concerning the link between obesity and diabetes. A majority of respondents possess fundamental knowledge about both obesity and diabetes, focusing on aspects such as typical blood glucose levels, fast food, soft drink consumption, and overall health implications. However, when delving into the root causes of these conditions, a notable knowledge gap exists. Respondents generally exhibit a favorable disposition towards diabetes associated with obesity. While the objective of maintaining a healthy weight is acknowledged, there appears to be a deficiency in awareness regarding the pivotal roles of physical activity and dietary practices in regulating body weight. The findings underscore the pressing need for a heightened emphasis on the development and implementation of educational initiatives. These programs should be designed not only to impart information but also to empower individuals to translate their knowledge and attitudes into actionable practices. By focusing on empowering individuals to effectively manage obesity-related diabetes, these education programs can play a pivotal role in addressing the prevailing challenges in this domain.

## 6 Limitations of the study

During the current study, majority of the data was self reported, which is susceptible to social desirability bias. Some errors of reporting in knowledge, attitudes, and practices may have occurred due to memory loss and reporting bias of the participants. These biases may have influenced the precisions and accuracy of the findings. Better results could be achieved by a more longitudinal approach to remove these biases in future studies. Some questions in the questionnaire didn't used person first language, that may have lead to the bias in the results.

## 7 Future recommendations

There is an obvious need for comprehensive and individualized educational programs designed to increase awareness and understanding of the complex relationship between obesity and diabetes. These programs should not only address the existence of these conditions, but also their causes, risk factors, preventative measures, and management strategies. Individuals can be empowered to make educated health decisions by creating engaging and culturally sensitive educational materials,. Targeted interventions should be designed to address the gaps in understanding among these groups. For instance, strategies could be tailored to address misconceptions, such as the notion that all individuals with obesity will develop diabetes. By customizing interventions to cater to different educational backgrounds, age groups, and socio-economic statuses, the impact of health education efforts can be maximized. Collaboration between healthcare providers, community organizations, and policymakers is crucial for effective health interventions.

## Ethics statement

The current study was approved by the Ethics Review Board of the Department of Biosciences, COMSATS University Islamabad (CUI), and it followed all ethical considerations. The reference number is CUI/Bio/ERB/06–18/23. A written informed consent was obtained from all the participants.

## Funding

This research did not receive any grant from funding agencies.

## Consent for publication

The informations were informed about the publication of the data obtained through questionnaire.

## Data availability statement

The data that support the findings of this study are openly available upon request.

## CRediT authorship contribution statement

**Arooj Kiran:** Methodology, Investigation, Formal analysis, Data curation, Conceptualization. **Naseer Ali Shah:** Writing – original draft, Validation, Supervision, Resources, Methodology, Investigation, Formal analysis. **Shujaul Mulk Khan:** Writing – review & editing, Validation, Investigation. **Haroon Ahmed:** Writing – review & editing, Visualization. **Muhammad Kamran:** Visualization, Data curation. **Beenish Khan Yousafzai:** Writing – review & editing, Conceptualization. **Zeeshan Ahmad:** Writing – review & editing, Visualization. **Sunghoon Yoo:** Writing – review & editing, Visualization, Funding acquisition. **Heesup Han:** Writing – review & editing, Visualization, Funding acquisition. **Ibrahim Alasqah:** Writing – review & editing, Visualization, Funding acquisition. **António Raposo:** Writing – review & editing, Visualization, Supervision, Funding acquisition.

## Declaration of competing interest

The authors declare that they have no known competing financial interests or personal relationships that could have appeared to influence the work reported in this paper.

## References

[1] Saleh F. (2012).

[2] Mohajan D., Mohajan H.K., J.J.o.I.i.M.R (2023).

[3] Faienza M.F. (2020).

[4] Sumińska M. (2022).

[5] Pérez-Bermejo M., Mas-Pérez I., Murillo-Llorente M.T. (2021).

[6] Al Kibria G.M., J.O.r. and c. practice (2019).

[7] Alfayez O.M. (2022).

[8] Hemming-Harlo M. (2019).

[9] Wang Z. (2021).

[10] Lovic D. (2020).

[11] Chamberlain J.J. (2016).

[12] Pantalone K.M. (2017).

[13] Moser J.A.S. (2019).

[14] Blüher M., J.N.R.E. (2019).

[15] Chobot A. (2018).

[16] Colagiuri S.J.D. (2010). Obesity and metabolism. Diabesity: Ther. Opt..

[17] Hu T. (2020).

[18] Gepstein V., Weiss R., J.F.i.e (2019).

[19] Peng L. (2021).

[20] Alam M.M. (2019).

[21] Al-Goblan A.S. (2014).

[22] Aljunid S.M. (2021). Laparoscopic Sleeve Gastrectomy.

[23] Manasa H., Mahadevaswamy R., J.S.J.N.H.C (2023).

[24] Di Cesare M. (2019). The epidemiological burden of obesity in childhood: a worldwide epidemic requiring urgent action. BMC Med..

[25] Ling J. (2023).

[26] Siddiqui M. (2018).

[27] Basit K.A. (2023). Changes in the prevalence of diabetes, prediabetes and associated risk factors in rural baluchistan; A secondary analysis from repeated surveys (2002–2017). PLoS One.

[28] Adnan M., Aasim M. (2020). Prevalence of type 2 diabetes mellitus in adult population of Pakistan: a meta-analysis of prospective cross-sectional surveys. Ann. Global Health.

[29] Abbas Q. (2023). Cognitive behavior therapy for diabetes distress, depression, health anxiety, quality of life and treatment adherence among patients with type-II diabetes mellitus: a randomized control trial. BMC Psychiatr..

[30] Aslam R. (2022). Pakistan: Prevalence, Trends and Management Strategies.

[31] Bukhsh A. (2020). Type 2 diabetes patients' perspectives, experiences, and barriers toward diabetes-related self-care: a qualitative study from Pakistan. Front. Endocrinol..

[32] Bibi S. (2021). Prevalence of obesity and impact of menopause on it among women of rural area of Punjab, Pakistan. Eur. J. Med. Health Sci..

[33] Martins R.B. (2021). Comparison of prevalence of diabetes complications in Brazilian and Mexican adults: a cross-sectional study. BMC Endocr. Disord..

[34] Su K.-z. (2019).

[35] Ganz M.L. (2014).

[36] Tong L. (2023).

[37] Kaštelan S. (2013).

[38] Mary G. (2014).

[39] Thondam S.K., Cuthbertson D.J., Wilding J.P. (2020).

[40] Kojta I., Chacińska M., Błachnio-Zabielska A.J.N. (2020).

[41] Wentzel A. (2022).

[42] Misra P.S. (2020).

[43] Bibi S. (2023). Knowledge, attitudes and practices regarding Taeniasis in Pakistan. Diseases.

[44] Raosoft I. (2020).

[45] Laar R.A. (2020).

[46] Shahid R. (2020).

[47] Tanzil S., Jamali T., J.J.A.M.C.A (2016).

[48] Jia H., Wang X., Cheng J., J.F.i.P.H (2022).

[49] ProCon B. (2020).

[50] Deng Q., Wei Y., Chen Y., J.F.i.P.H (2022).

[51] Azeem S. (2022).

[52] Soomro M.H., Jabbar A. (2024). BIDE's Diabetes Desk Book.

[53] Noreen Z. (2020). Transcriptional profiling and biological pathway (s) analysis of type 2 diabetes mellitus in a Pakistani population. Int. J. Environ. Res. Publ. Health.

[54] uddin Khand T. (2021).

[55] Khlaifat A.M. (2020).

[56] Han Y. (2023).

[57] Narayan K.M.V., Kanaya A.M. (2020). Why are South Asians prone to type 2 diabetes? A hypothesis based on underexplored pathways. Diabetologia.

[58] Narisada A. (2021). Sex differences in the association between fatty liver and type 2 diabetes incidence in non‐obese Japanese: a retrospective cohort study. J. Diabetes Investig..

[59] Lunger F. (2022). The impact of bariatric and metabolic surgery on cancer development. Front. Surg..

[60] Gadgil M.D. (2020). Circulating metabolites and lipids are associated with glycaemic measures in South Asians. Diabet. Med..

[61] Wang X. (2022).

