# Supplementary material for: Assessment of knowledge, attitude, and practices regarding the relationship of obesity with diabetes among the general community of Pakistan
Source: Heliyon. 2024 Apr 4;10(8):e29081. doi: 10.1016/j.heliyon.2024.e29081 (PMC11033058; doi:10.1016/j.heliyon.2024.e29081)
Supplement: Multimedia component 1 [file mmc1.docx]

Supplementary Table 1: Standard pre-validated questionnaire applied in the study.

### Sociodemographics:

- 1. Gender
     - Male
     - Female
  2. Age (years)
     - _________
  3. Religion
- _______
  1. Residence
- Urban
- Rural
  1. City/ village name
     - _________________
  2. Education
     - Illiterate
     - Primary
     - Middle
     - Matriculation
     - Intermediate
     - Graduate
     - Post-graduate
  3. Marital status
     - Married
     - Unmarried
  4. Family members
     - ________
  5. Weight (kg)
     - _______
  6. Hight
     - ______
  7. Occupation
     - Service
     - Housewife
     - Others_________
  8. Income/month
     - less than 10,000
     - less than 30,000
     - 31,000-51,000
     - 51,000-71,000
     - 71,000-91,000
     - above 91,000

### Knowledge:

- 1. Do you know the term “obesity”?
     - Yes
     - No
  2. Do you feel need to lose weight?
     - Yes
     - No
  3. Do you have the idea of the term “diabetes”?
     - Yes
     - No
  4. Are you a diabetic patient?
     - Yes
     - No
  5. Do you think obese patients have diabetes?
     - Usually
     - May be
     - May not
  6. Do you know obesity is associated with diabetes.
     - Yes
     - No
     - May be
  7. Obesity is also associated with hypertension, stroke, cardiovascular diseases, and some cancers?
     - Agree
     - Strongly agree
     - Disagree
     - Don’t know
  8. Have you ever heard the methods to determine obesity such as BMI, waist circumference and waist-hip ratio (WHR) etc.
     - Yes
     - Nos
  9. Do you know what is the ideal body weight?
     - Yes
     - No
  10. Do you know that a person with BMI 18.5-24.9 is considered healthy?
      - Yes
      - No
  11. Do you know a person with BMI 25-29.9 is considered overweight and a person with BMI 30.0+ is considered obese.
      - Yes
      - No
      - May be
  12. Do you know physical inactivity, eating too much fat, sleep routine, hormone disorders, stress, and anxiety, etc. are the causes of obesity?
      - Yes
      - No
      - May be
  13. Do you know how much energy require for ideal body weight?
      - Yes
      - No
  14. Do you think fast food, soft drinks, mayonnaise as healthier food for the body?
      - Yes
      - No
  15. Do you know having a blood glucose level less than 140 mg/dL (7.8 mmol/L) is normal?
      - Yes
      - No
  16. Do you know diabetes risk is highest in obese people with BMI 30 kg/m2?
      - Yes
      - No
  17. Eating unhealthy diet is one of the cause of diabetes?
      - Agree
      - Strongly agree
      - Disagree
  18. Obesity is believed to account for 80-85% of the risk of developing diabetes.
      - Agree
      - Strongly agree
      - Disagree e
  19. Prediabetes, also commonly referred to as borderline diabetes, is a metabolic condition in which the presence of blood glucose levels that are higher than normal but not yet high enough to be classed as diabetes, is closely related to obesity.
      - Agree
      - Disagree
      - Strongly agree
      - don’t know
  20. Obesity is also thought to trigger changes to the body’s metabolism, leading to reduced insulin sensitivity and, alternatively, developing into diabetes.
      - Agree
      - Strongly agree
      - Disagree
      - Don’t know

### Attitude:

- 1. Do you think obesity is a disease?
     - Yes
     - No
  2. Do you think normal body weight is important for good health?
     - Yes
     - No
     - May be
  3. Do you consider obesity a serious health problem?
     - Strongly agree
     - Agree
     - Disagree
     - Strongly disagree
  4. Do you think small weight loss can produce important health benefits such as lower the risk of developing diabetes, heart disease and types of cancer?
     - Yes
     - No
     - May be
  5. Do you think obese people must try to lose weight?
     - Yes
     - No
     - May be
  6. Do you think regular exercise helps you in losing weight?
     - Yes
     - No
  7. Have you ever tried to lose weight?
     - Yes
     - No
  8. Do you exercise daily?
     - Yes
     - No
     - occasionally
  9. Do you think being over-weight increase person's risk of developing diabetes?
     - Yes
     - No
     - May be
  10. Do you eat healthy diet?
      - Yes
      - No
  11. Do you care about your body shape?
      - Yes, very much
      - Not at all
      - Somewhat
  12. Do you think your body weight is under control?
      - Yes
      - No
      - May be
  13. In your opinion, family member of diabetic patient should aware regarding obesity?
      - Yes
      - No
      - May be
  14. Do you believe in precautionary measures to prevent the obesity and diabetes?
      - Agree
      - Strongly agree
      - Disagree
      - Neutral
  15. Do you believe diabetes is caused by obesity?
      - Agree
      - Strongly agree
      - Disagree
      - Neutral
  16. As long as diabetes is under control, there is no need to worry about diabetic complications.
      - Agree
      - Strongly agree
      - Disagree
  17. Do you believe in nondrug treatment of diabetes?
      - Yes
      - No
      - Somehow
  18. Are regular exercise increases the need for insulin or other medication for diabetes?
      - Yes
      - No
  19. Do you think the medication is more important than diet and exercise to control diabetes and obesity?
      - Agree
      - Strongly agree
      - Disagree
  20. Diabetic patients should take special care when cutting the nails of toes?
      - Yes
      - No

### Practices:

- 1. How frequently do you check your body weight?
     - Every 1-3 months
     - Every 4-6 months
     - Every 7-9 months
     - Every 10-12 months
  2. How many times have you tried to lose weight?
     - Once
     - Twice
     - More than twice
     - Never
  3. How do you tend to lose weight?
     - Gym
     - Home workout
     - Diet
     - Sports/ physical activities
     - None
  4. How much exercise do you do to lose weight?
     - 50 minutes a week
     - 100 minutes a week
     - 150 mins a week
     - 200 minutes a week
     - None
     - Others
  5. Do think weight counselling is a component of primary healthcare services?
     - Agree
     - Strongly agree
     - Disagree
     - Strongly disagree
  6. Have you ever visited the wight counselling seminar or workshop?
     - Yes
     - No
  7. Do you participate in any physical activity?
     - Yes
     - No
     - Sometimes
  8. What methods do you think are effective for your long-term weight loss?
     - Healthy diet
     - Physical activity
     - Exercise
     - No one
  9. How frequently you eat the junk food?
     - Once a day
     - Twice a day
     - More than twice a day
  10. Do you eat the sweet after meal?
      - Daily
      - Sometime
      - Rarely
  11. Do you eat more fruits and vegetables?
      - Yes
      - No
  12. Do you know karela water, jamun seeds, besan and Channa are effective in reducing blood sugar?
      - Yes
      - No
      - May be
  13. Do you test your blood for sugar?
      - Yes
      - No
  14. What method do you mainly use for testing your own sugar level?
      - Blood or urine test
      - Blood glucose test strips read by eye at home
      - Blood glucose test strips read by eye at home
      - Urine glucose test strips at home
      - None
  15. When your diabetes was first diagnosed, what treatment were you given?
      - Insulin
      - Tablets
      - Change Diet
      - Exercise
      - Lose weight
      - Other
      - None
  16. Do you experience any side effects of the medication that you know of?
      - Yes
      - No
  17. Do you take any additional nutritional supplements?
      - Vitamins
      - Herbal supplements
      - Others
      - None
  18. How often have you tested your sugar level in the last month?
      - Once a week or less
      - 2-6 times a week
      - Once a day
      - Never
  19. How often you visit a doctor for your diabetes?
      - 5 or more times year
      - 2- 3 times a year
      - Once a year
      - Never
  20. Do you think treating obesity-related diabetes is very costly?
      - Agree
      - Strongly agree
      - Disagree
      - Don’t know

## **Survey form link:**

- <https://forms.gle/h9UGJyYo8SqQqzrYA>

Supplementary Table 2: Socio-demographic characteristics of the respondents.

| **Variable** | **Category** | **Count (n)= 518** | **Frequency (%)** |
| --- | --- | --- | --- |
|  |  |  |  |
| Gender | Male | 275 | 53.1 |
|  | Female | 243 | 46.9 |
| Age | 18-24 | 191 | 36.9 |
|  | 25-34 | 144 | 27.8 |
|  | 35-44 | 61 | 11.8 |
|  | 45-54 | 71 | 13.7 |
|  | 55-64 | 31 | 6.0 |
|  | 65-74 | 20 | 3.9 |
| BMI | <18.5 | 81 | 15.6 |
|  | 18.5-24.9 | 317 | 61.2 |
|  | 25-29.9 | 89 | 17.2 |
|  | 30-39.9 | 31 | 6.0 |
| Education | Illiterate^$^ | 52 | 10.0 |
|  | Primary | 30 | 5.8 |
|  | Middle | 22 | 4.2 |
|  | Matric | 63 | 12.2 |
|  | Intermediate | 116 | 22.4 |
|  | Graduate | 205 | 39.6 |
|  | Post-graduate | 30 | 5.8 |
| Residency | Rural | 212 | 40.9 |
|  | Urban | 306 | 59.1 |
| Marital status | Married | 260 | 50.2 |
|  | Unmarried | 258 | 49.8 |
| Income/month | <10,000 | 12 | 2.3 |
|  | <30,000 | 154 | 29.7 |
|  | 31,000-51,000 | 198 | 38.2 |
|  | 51,000-71,000 | 86 | 16.6 |
|  | >71,000 | 68 | 13.1 |

^$^ The participant under “Illiterate” were those “Not attended any school”, while the rest were considered “Literate” in the analysis.
